# Immunomodulatory properties of dietary polyphenols: a role for combating infections at mucosal surfaces?

**DOI:** 10.20517/mrr.2025.81

**Published:** 2025-12-10

**Authors:** Mona Sharma, Yves Desjardins, Andrew R. Williams

**Affiliations:** ^1^Department of Veterinary and Animal Sciences, University of Copenhagen, Frederiksberg DK-1870, Denmark.; ^2^Institute of Nutrition and Functional Foods (INAF), Laval University, Québec City G1V 0A6, Canada.

**Keywords:** Polyphenol, mucosal immunity, immune response, gut microbiota

## Abstract

Polyphenols are food components with antioxidant and anti-inflammatory properties, and their health benefits are increasingly recognized in the context of noncommunicable diseases such as type 2 diabetes. However, their role in regulating immunity to infection is not well understood. Here, we highlight the various mechanisms by which polyphenols may enhance mucosal immunity via both adaptive and innate immune responses. Polyphenols may directly interact with host receptors on mucosal epithelial and/or immune cells to regulate production of cytokines and antimicrobial peptides. They can also modify gut microbiota composition, yielding microbial-derived metabolites that play a key role in fine-tuning immune function at mucosal surfaces. We provide examples of how these immunological changes may alter the outcome of pathogen infection and propose that an increased understanding of polyphenol-microbiota-immune interaction will provide a framework for the application of new nutrition-based strategies in the management and prevention of infectious diseases.

## INTRODUCTION

Several organs of the human body, including the intestines, urogenital tract, and respiratory tract, are lined with mucosa. Mucosal immunity serves as the first line of defense against infection, using antibodies of various isotypes and immune cells, such as tissue-resident lymphocytes, to fight against pathogenic microorganisms. Enhancing mucosal immunity, particularly in areas where pathogens are shed, can decrease infection rates, prevent the spread of novel variants, decrease disease severity, and help mitigate future pandemics^[1]^.

Polyphenolic compounds are secondary plant metabolites with over 8,000 known variants. They are widely found in various food items such as herbs, vegetables, seeds, fruits, nuts, olive oil, wine, and whole-grain cereals. These bioactive molecules have demonstrated numerous health benefits, including protection against cardio-metabolic and neurodegenerative diseases^[2]^. The action mode of polyphenols in mediating protection against these highly prevalent diseases is not completely understood but likely involves a combination of direct and indirect (prebiotic) mechanisms. Direct effects include the induction of anti-inflammatory pathways in diverse cell types (e.g., endothelial cells, enterocytes, and macrophages), thereby improving cardiometabolic health via downstream enhancements of blood vessel function and lowering low-density lipoprotein (LDL) cholesterol levels^[3]^. Prebiotic effects stem from the ability of dietary polyphenols and their metabolites to modulate gut microbiota (GM) composition towards an enterotype that may be associated with enhanced epithelial barrier function in the intestine^[2]^. However, the exact mechanisms that confer the health-promoting effects of polyphenols remain elusive. Moreover, while considerable research has focused on the role of polyphenols in protecting against noncommunicable metabolic diseases, their potential role in enhancing immunity to pathogens has received comparatively less attention. In an era of increasing antimicrobial drug resistance and future pandemic awareness, it is timely to consider the potential of polyphenols as dietary immune modulators that can aid infection resistance.

## POLYPHENOL STRUCTURE AND METABOLISM

**Figure 1:**
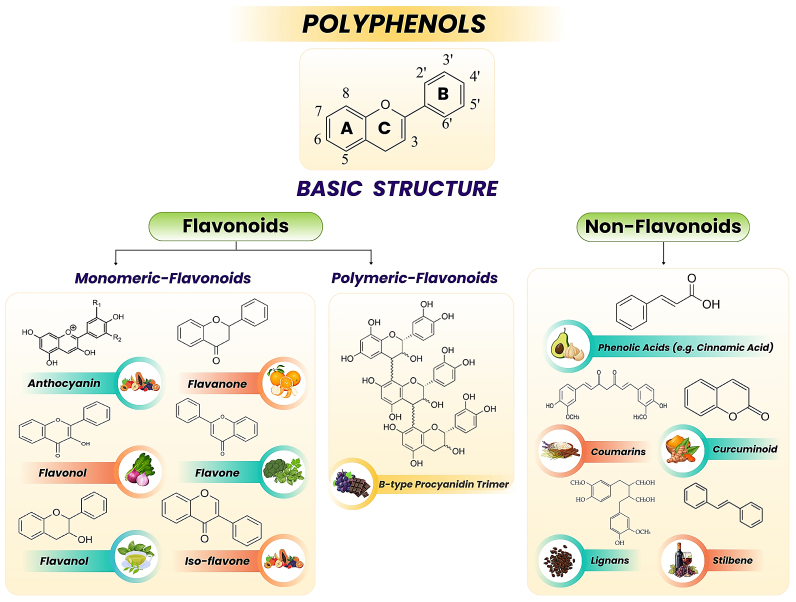
Example of common polyphenol structures. A basic polyphenol backbone consists of two aromatic rings (A and B) connected by a three-carbon bridge forming a heterocyclic ring (C). The figure includes examples of flavonoid and non-flavonoid monomers, as well as a polymeric procyanidin - a trimer composed of three flavan-3-ol units. The structures in the figure are the orginal work of the authors.

The structural basis of flavanols, present as either monomers or polymers, lies in a phenolic ring bearing one or more hydroxyl groups, a feature responsible for their potent free radical–scavenging and iron-binding activities^[4]^. Polyphenolic compounds are broadly categorized into two categories: flavonoids and non-flavonoids. Flavonoids comprise both monomeric forms - such as anthocyanins, flavonols, flavanols, flavanones, flavones, and isoflavones - and polymeric forms, including tannins such as proanthocyanidins and ellagitannins [Figure 1]. Non-flavonoids consist of a diverse range of compounds such as phenolic acids (e.g., benzoic and cinnamic acids), coumarins, stilbenes, quinones, lignans, and curcuminoids. These compounds are mainly present in complex forms - such as esters, glycosides, or high-molecular-weight polymers - which restrict their direct absorption in the small intestine. In their native form, polyphenols often require enzymatic hydrolysis or microbial transformation within the gastrointestinal tract to generate smaller, more bioavailable metabolites^[5]^. For instance, glycosylated flavonoids undergo deglycosylation by intestinal enzymes or GM before they are absorbed. Polymeric polyphenols such as proanthocyanidins are poorly absorbed in the upper gut and reach the colon, where they may interact with the microbiota and exert systemic biological effects.

### Metabolism of polyphenols

Most dietary polyphenols are poorly absorbed in the small intestine. Those that are absorbed - primarily small phenolic acids or non-glycosylated flavonoids such as catechin - undergo methylation, sulfation, and glucuronidation in enterocytes and hepatocytes before entering systemic circulation^[6]^. Unabsorbed polyphenols then move to the colon, where they undergo biotransformation, as gut microbes may utilize unabsorbed polyphenolic compounds as enzymatic substrates^[7]^. These microbes cleave the glycosidic linkage and break flavonol monomers and dimers, polyphenols into smaller metabolites; however, larger tannin-type polymers seem less amenable to degradation^[8]^. Polyphenol-derived metabolites are subsequently absorbed across the intestinal epithelium, subjected to phase I or phase II metabolism. They are either utilized or excreted in the serum or urine, or recycled back to the gut via enterohepatic circulation^[6,9-11]^. The glycosides of flavonoids are absorbed only after hydrolysis by gut microbes; however, anthocyanins may enter enterocytes through sodium-coupled glucose transporters in the small intestine without deglycosylation^[12]^. These polyphenol-derived metabolites have putative health benefits. For example, vanillic acid and 5-(3’,4’-dihydroxyphenyl)-γ-valerolactone, metabolites of peonidin and catechin, respectively, exhibit strong antioxidant activity, while protocatechuic acid, a metabolite of cyanidin, has anti-inflammatory properties^[13-15]^. Ellagitannin-derived metabolites such as urolithin A are also reported to be highly bioactive in cellular models^[16]^. In addition, phenolic acid metabolites bearing catechol and galloyl groups can exhibit iron-chelating properties and affect the growth of pathogenic bacteria^[17,18]^.

## MECHANISMS OF POLYPHENOL ACTION ON THE IMMUNE SYSTEM

The immune system consists of two principal components: innate immunity and adaptive immunity, each playing distinct roles. The innate immune system provides an immediate response to foreign antigens via various cellular and non-cellular mechanisms. Non-cellular innate defenses are based on localized immune responses to rapidly activate protective mechanisms, including the secretion of mucus, antimicrobial peptides, and enzymes. These effectors function to prevent pathogen entry, neutralize invading microbes, and facilitate their clearance from mucosal surfaces. Such defenses are particularly critical during the early stages of viral and parasitic infections, as they contribute to the establishment of a hostile microenvironment that restricts pathogen survival and distribution. The adaptive immune system, which includes T cells and B cells, is more specialized. It generates pathogen-specific antibodies and creates immunological memory for future encounters.

**Figure 2:**
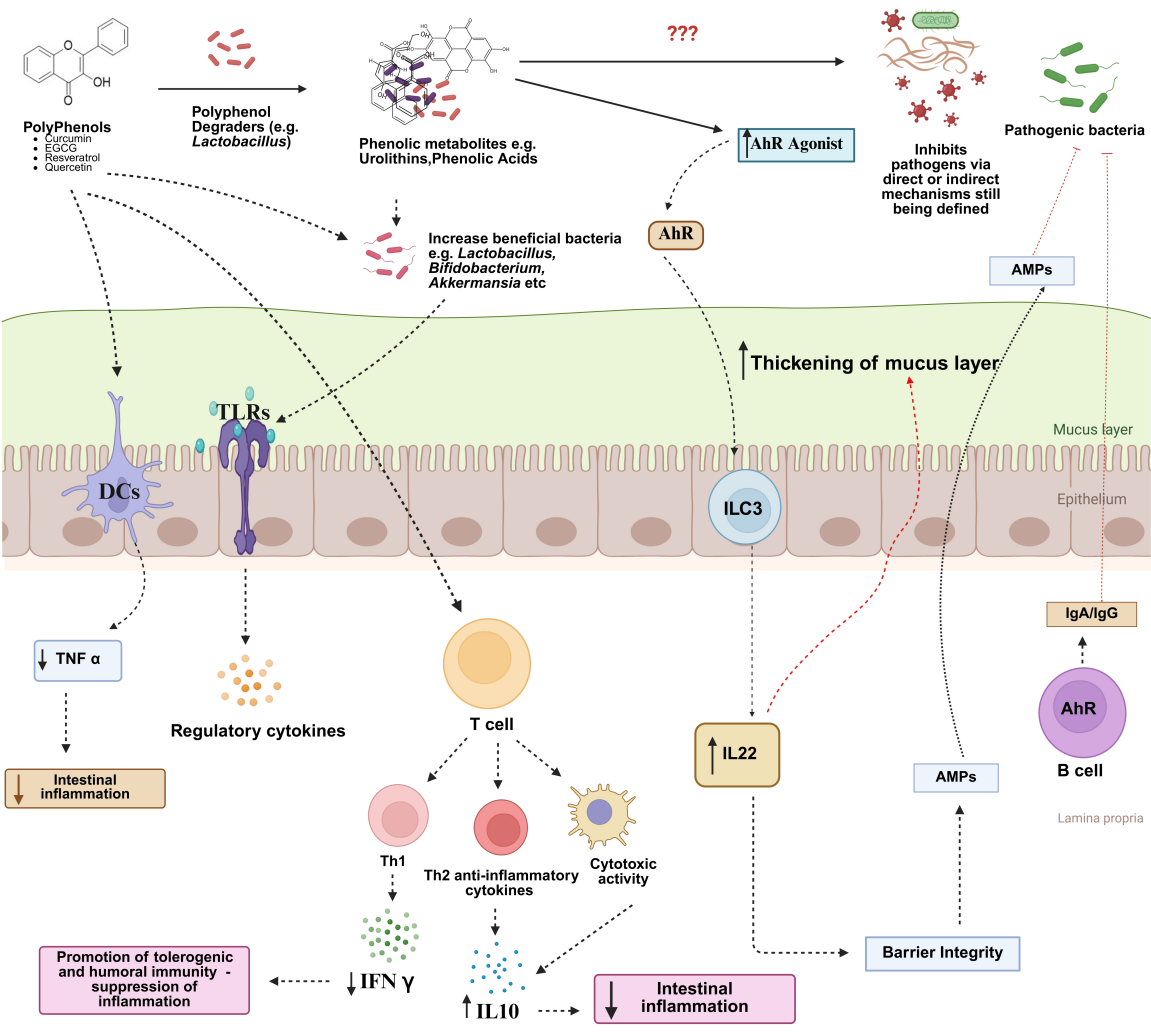
Possible mechanisms of how dietary polyphenols may modulate musical immunity to pathogens. Polyphenols may directly interact with dendritic cells or T-cells to alter cytokine secretion, changing the inflammatory tone with a reduction of pro-inflammatory cytokines such as IFNγ and promotion of Th2 and anti-inflammatory cytokines. Alternatively, polyphenols may change the composition of the gut microbiota, favoring the growth of bacteria such as *Akkermansia* spp. which can interact with toll-like receptors on epithelial cells to induce regulatory cytokine production. Finally, polyphenols may be degraded by certain gut microbes such as *Lactobacillus* spp. to yield phenolic metabolites which can potentially signal through the aryl hydrocarbon receptor (AhR) to promote production of IL22, antimicrobial peptides (AMPs), and mucosal IgA and IgG, which can all contribute to mucus production and immune-mediated neutralization of pathogens, whilst minimizing excessive inflammation and pathology. Created in BioRender. Williams, A. (2025) https://BioRender.com/zddbeiy. IL: Interleukin; IgA: Immunoglobulin A; IgG: Immunoglobulin G; Th2: T helper type 2 cells; Th1: T helper type 1 cells; IFN-γ: Interferon-γ; TNF: tumor necrosis factor; ILC3: group 3 innate lymphoid cells.

The role of diet and the GM in regulating mucosal immune function is becoming increasingly evident. Immune cells are highly responsive to both dietary components (e.g., vitamins) and microbiota-derived metabolites, including short-chain fatty acids (SCFAs) and indole derivatives^[19]^. These molecules influence immune cell differentiation, cytokine production, and barrier integrity. Therefore, nutritional interventions aimed at modulating the GM and enhancing the production of beneficial microbial metabolites represent a promising strategy to support mucosal immunity, prevent infections, and reduce reliance on antimicrobial drugs. However, whether dietary polyphenols contribute to improved immune defense against infection remains equivocal. Here, we propose a potential framework describing how polyphenols may regulate the immune system [Figure 2] and how this could be leveraged to reduce pathogen burdens.

### Direct effects of polyphenols on immune cells

#### Intestinal epithelial cells

As the initial point of contact between dietary constituents and the mucosal immune system, intestinal epithelial cells (IEC) may play a vital role in transmitting the immunomodulatory effects of polyphenols. The metabolic function of IEC is substantially modified during inflammatory or stress events, leading to heightened production of reactive oxygen species (ROS) and altered bioenergetics (increased glycolysis usage). These inflammation-induced events are markedly attenuated by polyphenols in *in vitro* models^[20]^. Epithelial cell-derived cytokines such as thymic stromal lymphopoietin are also regulated by polyphenols in allergic inflammation^[21]^. Notably, polyphenols also induce goblet cell hyperplasia (leading to increased mucus production) in animal models of obesity and colitis^[22]^. Mechanistically, polyphenols (e.g., grape-derived procyanidins) may drive mucus production by remodeling of the cellular composition and function of the epithelium, as indicated by organoid models where polyphenols specifically triggered L-cell proliferation and goblet cell makers such as Mucin 2 (Muc2)^[23]^. The receptor(s) that recognize polyphenols are not well-defined; however, polyphenol metabolites such as urolithin A can trigger thickening of the mucus layer via the aryl hydrocarbon receptor (AhR)-NF-E2-related factor 2 (Nrf2) pathway^[24]^, and AhR activation by chemically diverse polyphenols in cell lines has been reported^[25]^. AhR is a transcription factor highly expressed in mucosal barrier tissues and immune cells. It senses xenobiotics from the diet or GM, linking metabolite detection to immune surveillance and function, and may thus play a key role in mediating the immunomodulatory effects of dietary polyphenols^[26]^. In addition, polyphenols may influence multiple intestinal cell types beyond the epithelium. For instance, they can modulate enteric neurons and key components of the gut-brain axis, affecting gastrointestinal motility and function^[27]^. However, the direct effects of polyphenols on other intestinal cell types remain less well established and further studies are needed to elucidate these interactions and their potential roles in modulating the immune response.

#### Dendritic cells and macrophages

Dendritic cells (DCs) play an important role in initiating and regulating innate immune responses by presenting pathogen antigens to naïve T-cells in lymphoid tissues. The cytokine milieu that accompanies this initial DC/T-cell interaction is critical in shaping subsequent adaptive immune responses; therefore, modulators of DC activity may play key roles in immune regulation. Polyphenols directly affect DCs in terms of differentiation, maturation, cytokine secretion, and antigen presentation^[28,29]^. Exposure of DCs to quercetin or Epigallocatechin-3-Gallate (EGCG) leads to downregulation of major histocompatibility complex class II (MHC II) molecules and integrin alpha X (CD11c), cluster of differentiation 80 (CD80), and cluster of differentiation 83 (CD83), preventing full maturation, and consequently driving an anti-inflammatory T-cell phenotype^[30-32]^. Consistent with this, proanthocyanidins are endocytosed by human DCs, which leads to suppression of interleukin (IL)-12p70 and IL-6 secretion and the production of interferon-γ from DC-activated T-cells^[33]^. However, the secretion of IL-10 was seen to increase, with these effects associated with an increasing degree of polyphenol oligomerization^[33]^. Thus, a consistent feature of DC-polyphenol interactions is the promotion of a tolerogenic phenotype, which may dampen subsequent inflammatory responses.

Macrophages are specialized cells derived from monocytes and play a crucial role in tissue repair and immune defense^[34]^. They are classified into two phenotypes: the M2 phenotype, characterized by anti-inflammatory, anti-parasitic, and wound-healing properties, and the M1 phenotype, which exhibits pro-inflammatory properties. Polarization towards M1 is initiated by bacterial lipopolysaccharides and interferon-γ, whereas IL-4 induces M2 polarization^[35]^. Cocoa polyphenols reduce M1-driven inflammation and promote M2 polarization in activated macrophages^[36,37]^. Moreover, the medicinal plant *Innotas sanghuang*, which contains rutin, quercetin, isorhamnetin, and chlorogenic acid, reduces inflammation by modulating macrophage-adipocyte interactions and facilitating recovery from insulin resistance and metabolic syndrome^[38]^. Quercetin has also been reported to suppress IL-6, IL-1β, and tumor necrosis factor-α (TNF-α) secretion^[39]^. Similarly to DCs, the structure of polyphenol-based oligomers (i.e., proanthocyanidins) has a significant impact on their role in regulating inflammatory cytokines; proanthocyanidin polymers induce substantial transcriptomic changes in macrophages, with dimers having far less impact^[40]^. Collectively, these studies indicate that polyphenols have structure-dependent modulatory activity on DC and macrophage function, which may have profound implications for responses to infection at mucosal barriers.

#### B and T cells

Polyphenols have also been linked to the modification of enzymatic signaling by blocking tyrosine-protein kinase and serine-threonine pathways. These enzymes are primarily associated with the proliferation of T cells, activation of B cells, and cytokine production by activated monocytes^[41]^. Tregs are responsible for immune tolerance and the regulation of autoimmunity. Rats treated daily with 10 mg/kg body weight of EGC-M5 - a major metabolite of epigallocatechin gallate - for 14 days exhibited enhanced cytotoxic activity of natural killer (NK) cells and improved functional responses of CD4^+^ T cells^[42]^. Polyphenols have also been shown to enhance the production of IL-10 in human cell culture^[43]^. Epigallocatechin gallate from green tea has been shown to decrease the migration and adhesion of CD8^+^ T cells by downregulating the expression of cluster of differentiation molecule 11B (CD11b); hence, it serves as an anti-inflammatory agent^[44]^.

### Immune-modulation through polyphenol-mediated shifts in gut microbiota composition

GM has great importance in maintaining healthy physiological functioning, and diet plays a major role in governing its composition. Dietary polyphenols have been shown to influence the GM significantly through various mechanisms. *In vitro*, red wine flavonoids can inhibit the growth of *Clostridium histolyticum*^[45]^, while increasing the growth of *Enterococci* and *Bifidobacterium*. The growth of *Bifidobacterium* was also enhanced by gingerol from ginger, tannin from pomegranate, and sorghum polyphenols^[46]^. Other studies have shown that the phenolic compounds regulate the intestinal micro-ecosystem by enhancing the growth of beneficial bacteria such as *Akkermansia, Faecalibacterium, Lactobacillus, Bifidobacterium,* and *Enterococcus spp*^[47]^*. In vivo*, polyphenol-rich mango supplements prevent the loss of beneficial intestinal bacteria such as *Akkermansia*, *Aldercreutzia,* and *Bifidobacteria* when mice are fed a high-fat diet^[48]^. Polyphenol-rich cranberry extract, which is rich in proanthocyanidins and flavonoids, also enhances *Akkermansia* growth and decreases the ratio of *Firmicutes* to *Bacteroides* in obese mice^[49]^. Studies with human volunteers have also demonstrated the beneficial effects of polyphenols on the GM^[50]^. Anthocyanin-rich blueberries increased *Bifidobacterium* and lactic acid bacteria in healthy volunteers^[51]^. In the same way, the incorporation of almonds or almond skin in the diet of human volunteers showed an increase in *Bifidobacterium* and *Lactobacillus* in the faeces^[52]^.

**Figure 3:**
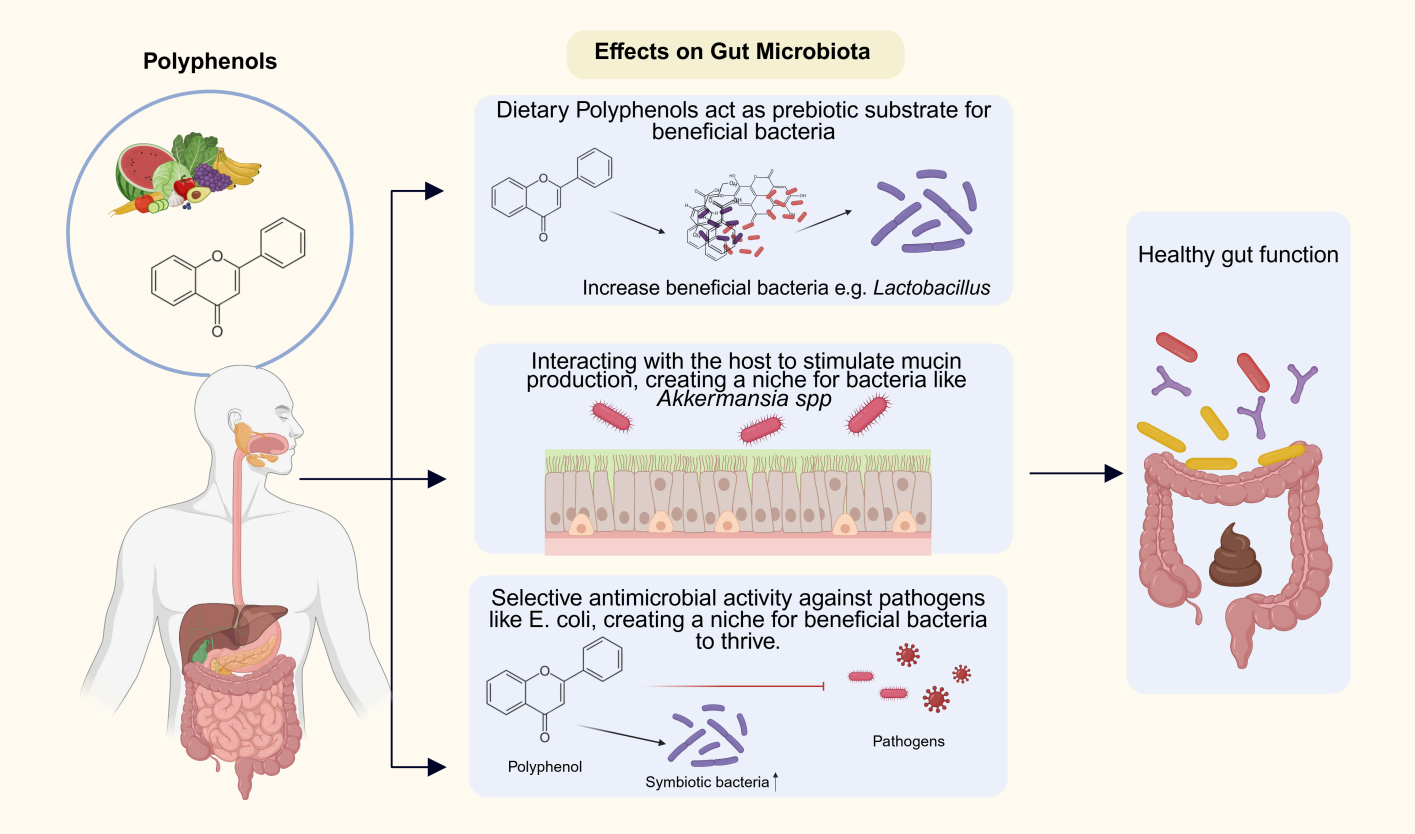
Polyphenol-mediated Regulation of Gut Microbiota. Dietary polyphenols derived from fruits and vegetables exert multiple beneficial effects on gut microbial composition. Polyphenols may act as prebiotic substrates that enhance the growth of beneficial bacteria, such as *Lactobacillus*. They may interact with the host epithelium to stimulate mucin production, supporting the proliferation of mucin-utilizing bacteria, including *Akkermansia* spp. Finally, polyphenols exhibit selective antimicrobial activity against pathogenic bacteria (e.g., *E. coli*), thereby creating a favorable niche for symbiotic bacteria. Collectively, the changes in gut microbiota composition can calibrate the activity of immune cells residing at the mucosal barrier. Created in BioRender. Williams, A. (2025) https://BioRender.com/2z1gbuw.

As shown in Figure 3 polyphenol-mediated effects may stem from reciprocal effects of (1) acting as a prebiotic substrate which can be utilized by putatively beneficial bacteria such as *Lactobacillus* spp.; (2) interacting with the host to stimulate the mucin production, thus creating an ecological niche for bacteria such as *Akkermansia* spp.; and (3) selective antimicrobial effects against pathogenic bacteria such as *Escherichia coli* (*E. coli*), forming a niche for advantageous bacteria to thrive^[53]^.

Changes in GM composition are often associated with alterations in immune function at mucosal surfaces, such as modified cytokine production, improved epithelial barrier integrity, and Immunoglobulin A (IgA) secretion^[54,55]^. The dampening of inflammation may be a key factor linking the consumption of polyphenols to improved health outcomes in obesity or diabetic mice. Microbiota transfer studies provide evidence for a causal involvement of the GM in the protective actions of polyphenols, reproducing the beneficial effects of polyphenol consumption in obesity^[56]^. Mechanistically, *Akkermansia muciniphila* contains unique parietal lipooligosaccharides^[57]^ which can directly modulate the inflammatory tone at barrier surfaces, as they can signal through receptors such as Toll-like receptor 2 (TLR2) to promote regulatory cytokine production, in contrast to bacteria such as *E. coli,* which induce Toll-like receptor 4 (TLR4)-driven pro-inflammatory responses^[57]^. However, GM-mediated activity of polyphenols may not simply arise from the growth of different bacterial taxa. As mentioned above, polyphenols can be extensively metabolized by GM to yield metabolites with significant bioactivity. Some of these, e.g., valerolactones, Urolithins A and B, and flavonoid derivatives, have been implicated in the beneficial effects of dietary polyphenols on metabolic disorders^[58-60]^. Notably, microbial-derived metabolites from polyphenols are potent modulators of human T-cell activity^[61]^, and can also alter T-helper cell responses in murine models of chronic stress^[62]^. Given the potent immune-modulating effects of other GM-derived molecules such as SCFA, further studies are needed to elucidate how polyphenol-derived metabolites influence the activity of innate and adaptive immune cells.

## A PROTECTIVE ROLE FOR POLYPHENOLS IN IMMUNITY TO PATHOGENS?

Mucosal immune function is an intricately orchestrated network that can generate diverse and precisely calibrated responses to different infectious agents. The robust immunomodulatory properties of polyphenols raise the possibility that their consumption may alter immunity to mucosal pathogens. Whilst polyphenols have strong anti-inflammatory properties that may aid in the resolution of chronic disease, how they influence specific responses to pathogens requires careful consideration.

Polyphenols have shown promise for combating respiratory pathogens such as influenza and coronaviruses. In humans, apple-derived procyanidins can boost specific antiviral responses, e.g., neutralizing antibody production or production of type-1 interferons from monocytes^[63]^, which may aid in limiting the initial viral replication. Moreover, their anti-inflammatory properties, such as modulation of macrophage polarization as described above, may have beneficial effects by muting harmful inflammation that drives the pathology associated with, for example, coronavirus disease 2019 (COVID-19). Polyphenols additionally exercise antiviral activities by targeting viral proteins, blocking cellular receptors, and preventing viral replication^[64,65]^. Taken together, these data suggest that polyphenols may boost pathogen-specific immunity in the respiratory tract while limiting non-specific immunopathology, which damages host tissues. A similar situation may occur during gastrointestinal infection. Apple polyphenol extracts protect against *Clostridioides difficile* (*C. difficile*) by enhancing survival rates through reducing diarrhoea symptoms and modulating the GM^[66]^, and diverse polyphenol compounds can enhance the eradication rates of *Helicobacter pylori*^[67,68]^. A consistent feature of the immunomodulatory effects of polyphenols during intestinal infection is enhanced production of mucosal antibodies and antimicrobial peptides, and suppression of oxidative stress markers and inflammatory cytokine production^[69-72]^. These findings suggest that polyphenols promote a specific immunological tone in mucosal tissue, whereby production of specific antimicrobial immune components is favored, and non-specific inflammation is suppressed. Potentially, AhR-activation by dietary polyphenols may contribute to this immunotype, as AhR-driven IL-22 production aids calibration of the immune tone at barrier surfaces and restrains inflammatory responses.

Despite their beneficial effects, polyphenols may, under certain conditions, exacerbate infection. In murine models, incorporation of polyphenols into synthetic diets has been shown to increase susceptibility to infection with Gram-negative bacteria or to amplify dysbiosis associated with parasitic infection in the cecum^[73,74]^. Therefore, important context-dependent interactions may exist between polyphenols, the food matrix in which they are incorporated, and host immunity to infection. Furthermore, the bacteria that thrive within the GM during polyphenol consumption can potentially have deleterious effects. For example, whilst *Akkermansia* is associated with numerous health benefits, it may aggravate food allergies^[75]^. In addition, *Eggerthella lenta* (*E. lenta*) is a microbe that (similar to *Lactobacillus plantatum*) can metabolize flavan-3-ols and increases in abundance following polyphenol intake. However, *E. lenta* can also increase respiratory pathogen infection by changing bile acid metabolism, leading to altered neutrophil function in the lung and impaired host defense^[76]^. More research is clearly required to understand polyphenol-microbiota-immune interplay and how this relates to infection resistance. Thus, whilst polyphenols may generally drive an immune network in mucosal tissues that aids the expression of pathogen-specific immunity, each different host-pathogen system may require empirical investigation to determine if polyphenols have beneficial effects.

## FUTURE DIRECTIONS

### Polyphenols as dietary supplements to boost mucosal immunity

Infectious diseases will continue to be a major issue in the 21st century due to critically high rates of antimicrobial drug resistance. Large challenges exist, ranging from *C. difficile* in many high-income countries to persistent bacteria and parasitic infections (e.g., rotavirus, hookworm, *Giardia*), in low- and middle-income countries together with rapidly urbanizing societies such as China, Brazil, and Africa. Preventing infection by leveraging dietary components that enhance mucosal immunity and strengthen barrier defenses in the gut or other mucosal surfaces represents an untapped opportunity to relieve infection pressure and thereby preserve antimicrobial drug efficacy. The immunomodulatory properties of polyphenols, while increasingly understood in the context of noncommunicable diseases, remain relatively unexplored in the context of infection and immunity. However, it is now emphasized that the suppression of ROS and inflammatory cytokines by dietary polyphenols may concomitantly boost mucosal antimicrobial peptides and antibodies such as IgA, which can limit infection. Moreover, as the production of inflammatory mediators and ROS can be co-opted by pathogens such as *Salmonella* to enhance their survival in the host^[77]^, the antioxidant properties of polyphenols may play a key role in regulating the gut ecosystem to limit the niche that pathogens exploit to ensure their persistence in the body.

### Challenges and research gaps

Several key questions remain unresolved. The mechanisms by which polyphenols exert their immunomodulatory effects are still largely unknown. Are polyphenols metabolized by gut bacteria into small metabolites that can signal through receptors such as the AhR to modulate immune cell activity? Are there sentinel cells (e.g., tuft cells) responsible for sensing phenolic metabolites and relaying signals to leukocytes, or do polyphenols signal more directly, altering DC or macrophage activity? In addition to these basic mechanistic studies, there is a need for large-scale clinical trials and population studies to determine the therapeutic usefulness of diverse polyphenolic compounds. The lack of standardized procedures hampers the synthesis of polyphenolic conjugates with health-promoting and functional properties. Establishing a standardized approach would greatly benefit the food and pharmaceutical industries. More studies are needed to prove their practical usage in formulations, hence encouraging widespread use and integration^[78]^. To elucidate the molecular mechanisms of polyphenols, human trials and multidisciplinary approaches (such as bioinformatics and molecular techniques) should be used to validate the benefits and for a better understanding of various factors. Moreover, while polyphenol-rich dietary supplements offer numerous health benefits, they can also have adverse effects, particularly when consumed in high doses or over prolonged periods. Reported side effects include gastrointestinal discomfort such as nausea and diarrhea, especially with compounds such as resveratrol and catechins^[79]^. Additionally, polyphenols may interfere with iron absorption, leading to deficiency disorders^[80]^. Therefore, further studies are required to better elucidate both the benefits and risks associated with polyphenol intake. Future research should also investigate the synergistic interactions of polyphenols with other dietary components and probiotics, as well as their potential roles in protecting against infectious diseases. Such investigations could pave the way for innovative polyphenol-based therapeutic strategies aimed at strengthening mucosal defenses and improving overall health outcomes.

## CONCLUSION

The health-promoting properties of polyphenols are now well recognised, particularly within non-communicable diseases. Emerging evidence suggests that the immune response to infection may also be modulated by polyphenols. The relative contributions of direct and indirect (i.e. via the gut microbiota) immunomodulatory actions remain to be established. Immunity at mucosal barriers is fine-tuned to protect against different pathogens, and thus it is not necessarily the case that polyphenol-mediated immune changes will protect against all infections – indeed, in some cases infection could be exacerbated. Thus, a rigorous mechanistic understanding of the interplay between polyphenol-rich dietary components, the gut microbiota, and the immune system is required. Such knowledge should allow polyphenol-based precision nutrition approaches to target the mucosal immune system and help slow the spread of antimicrobial drug resistance.

## DECLARATIONS

### Copyright

© The Author(s) 2025.
